# Understanding and responding to remote mental health help-seeking by gay, bisexual and other men who have sex with men (GBMSM) in the U.K. and Republic of Ireland: a mixed-method study conducted in the context of COVID-19

**DOI:** 10.1080/21642850.2022.2053687

**Published:** 2022-04-05

**Authors:** Dimitra Eleftheria Strongylou, Paul Flowers, Ruth McKenna, Ross Andrew Kincaid, Dan Clutterbuck, Mohamed Ahmed Hammoud, Julian Heng, Yvonne Kerr, Lisa McDaid, Jamie Scott Frankis

**Affiliations:** a School of Health and Life Sciences, GCU, Glasgow, UK; b Department of Psychology, University of Strathclyde, Glasgow, UK; c Waverley Care, Edinburgh, UK; d Chalmers Centre, NHS Lothian, Edinburgh, UK; e Kirby Institute, UNSW, Sydney, Australia; f Public Health Department, NHS Greater Glasgow and Clyde, Glasgow, UK; g Public Health and Health Policy Department, NHS Lothian, Edinburgh, UK; h Institute for Social Sciences Research, The University of Queensland, Brisbane, Australia

**Keywords:** Mental health, COVID-19 recovery, GBMSM, behaviour change wheel, gay and bisexual men

## Abstract

**Background:** Gay, bisexual and other men who have sex with men (GBMSM) are at far greater risk of experiencing poor mental health (MH) than wider society. This disparity was exacerbated by additional ‘unique to sexual minority status’ COVID-19 stressors.

**Objective:** This sequential, mixed-methods study examined remote MH help-seeking among GBMSM in the U.K. and Ireland during the first COVID-19 lockdown.

**Methods and Results:** Quantitative survey data (*n* = 1368), analysed with logistic regression, suggested GBMSM experiencing moderate-to-severe anxiety and those with a past MH diagnosis were most likely to seek MH support. Thematic analysis of qualitative interview (*n* = 18) data identified multiple barriers and enablers to GBMSM seeking remote MH help, with the help primarily sought from GBMSM-facing organisations and generic online resources. Finally, the behaviour change wheel was used to generate theoretically informed recommendations to promote MH help-seeking among GBMSM in Scotland.

**Implications:** We discuss how applying these recommendations in the short, medium and long term will begin to address GBMSM’s MH needs, post COVID-19.

## Introduction

Our attempts to understand and mitigate the harms caused by COVID-19 are only just beginning. Herein, we consider how COVID-19 impacted one, often marginalised, group and appears to have exacerbated previously established health inequalities. We explore how mental health (MH) help-seeking amongst gay, bisexual, and other men who have sex with men (GBMSM) was shaped by the impact of COVID-19 during the U.K.’s first lockdown. To take forward recovery and build resilience within these communities, we begin the process of learning from COVID-19-related experiences both to conceptualise and plan services differently and address the long-standing inequalities affecting GBMSM. Then we use our findings to develop preliminary theory-based recommendations to reduce long-standing inequalities in GBMSM’s health.

### GBMSM, MH, and COVID-19

Before COVID-19, GBMSM were disproportionately affected by multiple health inequalities compared to the wider population, including poorer MH (McDaid et al., 2020; McGarty et al., 2021). For instance, according to an Australian study, before COVID-19, 18% of all GBMSM were suffering from moderate-severe anxiety, 28% from moderate-severe depression, and half reported use of illicit drugs in the previous six months (Prestage et al., 2018). During COVID-19, further impacts upon GBMSM’s health have been documented, including a rise in levels of anxiety, depression, and loneliness (Frankis, Strongylou, & Kincaid, 2020; Holloway et al., 2021). In an Australian online cohort study of gay and bisexual men, the proportion of participants with moderate-severe depression increased from 19% to 26%, while the proportion of participants with moderate-severe anxiety increased from 13% to 17% (Bavinton et al., 2022). Indeed, sexual minorities reported greater psychological stress, depression, and anxiety associated with the pandemic than sexual majorities (Moore, Wierenga, Prince, Gillani, & Mintz, 2021; Peterson, Vaughan, & Carver, 2020). This is because additional, sexual minority-specific stressors (e.g. reduced connection to the LGBT community, sexual orientation-related family conflict etc.) further impacted LGBT people's MH, in addition to those generic COVID-19 stressors (Suen, Chan, & Wong, 2020) experienced by all. Thus, while studies have focused upon GBMSM’s MH during the COVID-19 pandemic, less is known about their MH help-seeking behaviours.

### MH help-seeking behaviour in GBMSM

The high prevalence of mental health problems among GBMSM is mismatched with their efforts to seek mental health support. Despite the growing research in the area, there is lack of consensus on the definition of MH help seeking, given the complexity of the construct. Rickwood et al. (2012) explain that ‘[mental health] help-seeking is an adaptive coping process that is the attempt to obtain external assistance to deal with a mental health concern’. MH support can be sought from several sources, specifically, informal (e.g. friends, family), formal (e.g. MH professionals), semi-formal (e.g. other health professionals such as GPs, nurses, support workers), and self-help (e.g. MH apps, books, CDs) sources (Kauer, Mangan, & Sanci, 2014; Xu et al., 2018). Rickwood et al.’s (2005) model proposes that MH help-seeking behaviour requires: (1) an initial self-awareness of a MH problem, (2) a recognition of both symptoms and the need for support, (3) the identification of accessible sources of support, and, finally, (4) a willingness to seek support and disclose relevant information where necessary.

Before COVID-19, MH help-seeking behaviour was widely examined in a number of groups, including young people (Divin et al., 2018; Kauer et al., 2014; Pretorius, Chambers, & Coyle, 2019), African American youth (Planey, Smith, Moore, & Walker, 2019), men (Galdas, Cheater, & Marshall, 2005), rural residents (Cheesmond, Davies, & Inder, 2019), and the armed forces (Coleman, Stevelink, Hatch, Denny, & Greenberg, 2017). Systematic review evidence suggests that MH help-seeking rates are relatively low among people with diagnosed mental health issues, with around one-third seeking help in the last year (Gulliver, Griffiths, & Christensen, 2010). However, there is a scarcity of research examining MH help-seeking behaviour among GBMSM and none during COVID-19. Generally, GBMSM-related MH help-seeking research has predominantly focused upon identity-specific barriers that exist among GBMSM when seeking help for their MH (McDermott, 2015; Wu & Lee, 2021; Zay et al., 2021) such as LGBTQ stigma and a dual LGBTQ MH stigma (McDermott, 2015; Zay et al., 2021). However, since the social distancing requirements of COVID-19 shifted most health services to online (websites) or telehealth delivery (Murphy, 2020), we now need to understand this remote MH help-seeking, which has increased 12-fold during the pandemic (Pierce, Perrin, Tyler, McKee, & Watson, 2021).

### Remote MH help-seeking

While websites have offered therapeutic MH resources since the late 1990s, the quality of information provided is unregulated and so may be lacking (Reavley & Jorm, 2011) or biased (Read & Cain, 2013). Despite this, several websites, mostly drawing on cognitive behavioural therapy (CBT) and mindfulness, have been evaluated as being effective (Rogers, Lemmen, Kramer, Mann, & Chopra, 2017). More recently, meta-analyses and systematic review evidence suggest that specific smartphone apps (again primarily drawing on CBT-derived therapy (e.g. Woebot) or mindfulness-based meditation (e.g. InsightTimer, Headspace)) can reduce stress, depression, anxiety and improve psychological well-being (Firth et al., 2017a, 2017b; Gál, Ștefan, & Cristea, 2021; Rathbone, Clarry, & Prescott, 2017). In an Australian study, it was found that among young men with MH issues, talking online helped (81%), and these men were also satisfied or very satisfied with the online help they received (83%) (Collin et al., 2011). Similarly, crisis phone-lines have been assessed as cost-effective and easy to access compared to face-to-face services (Bradford & Rickwood, 2014) and are effective in relation to several MH-related outcomes including enhanced mood and prevention of self-directed violence (Hoffberg, Stearns-Yoder, & Brenner, 2020).

However, the user’s ability to identify scientifically robust websites, apps and phone lines is unknown (Rogers et al., 2017). This has led health psychologists to examine how individuals seek support from services available to them.

### MH services in the U.K. and Republic of Ireland

Within the U.K., health is a devolved matter, meaning each constituent country sets its own agenda to approach MH. Mental health policy of the four U.K. countries and The Republic of Ireland is largely similar; each country’s MH strategy focusses on prevention and early intervention, explicitly recognising inequalities relating to sexuality, alongside other characteristics (Dean, 2017). This is underpinned by MH services delivered by nationwide governmental health services and various non-governmental organisations. For example, in Scotland the NHS and several Scotland- (e.g. SAMH, Breathing Space) and U.K.-wide (e.g. Mental Health Foundation, MIND) charities deliver MH services on a population-wide basis, the charity LGBT Health and Wellbeing provide LGBTQI+ people with specialised MH support, whilst GBMSM-facing^1^ sexual health services and additional sexual health / blood-borne viruses charities (i.e. Waverley Care/S-X, Terrence Higgins Trust and HIV Scotland) offer holistic services engaging with GBMSM about their wider mental and physical health, albeit within a sexual health and HIV prevention context. Services include online information and self-help guides, telephone, online-chat and group-based support, GP pharmacological, brief-intervention, and individual counselling/ psychological support, provided through a combination of remote and in-person delivery. MH service provision adopts a largely similar model in the remaining U.K. countries and The Republic of Ireland, although availability, service waiting times and level of LGBTQI+ tailored support varies both within and across different countries.

### Research questions

As well as shifting most semi-formal and formal sources of support remotely, COVID-19 lockdown removed many *informal* sources of support for GBMSM (e.g. commercial gay scene, sexual contacts, face-to-face meeting with friends). This study aims to address the sources of Rickwood et al.’s MH help-seeking model (2005); formal and self-help sources; in the present study formal sources primarily relate to gay facing organisations facing organisations whilst self-help sources concern the remote help-seeking described above. Accordingly, we examine GBMSM’s MH help-seeking behaviours during the first COVID-19 lockdown (Mid-March to End June, 2020), to develop evidence and theory-based recommendations for service providers by addressing the following research questions (RQ).

**RQ1:** What are the key sociodemographic and psychological factors predicting remote MH help-seeking among GBMSM during the first COVID-19 lockdown in the UK and Republic of Ireland?

**RQ2:** What are the barriers and facilitators to remote MH help-seeking among GBMSM in Scotland?

**RQ3:** What are the preliminary recommendations to enable services to enhance MH help-seeking among GBMSM?

## Methods

**Figure 1.**
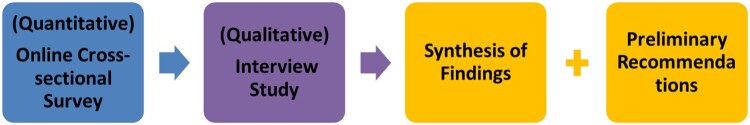
The sequential mixed-methods design employed in the current study.

**Figure 2.**
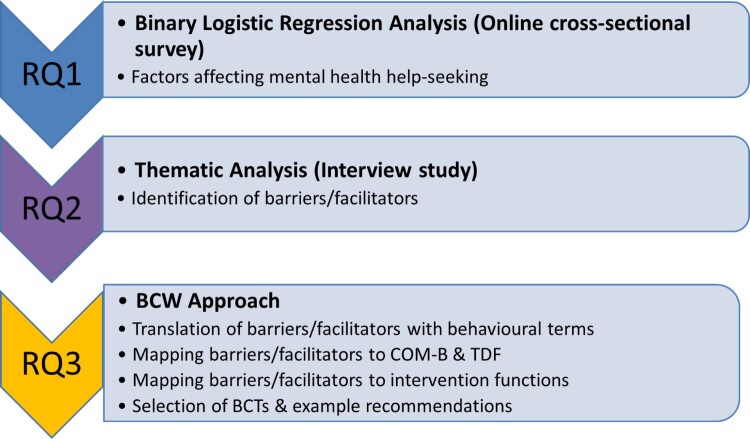
Analytical steps taken to address RQ1, RQ2 & RQ3.

A sequential mixed-methods design was employed (see Figure 1), where online quantitative data analysis informed qualitative data theoretical sampling. Subsequently, findings were categorised and subjected to further behavioural analysis to provide holistic recommendations to improve MH help-seeking among GBMSM (Figure 2 summarises the analytical steps taken to address our three research questions).

### Quantitative study

The Social Media, Men who have sex with men, Sexual and Holistic Health – Pandemic study (SMMASH-Pan) collected anonymous, self-completed questionnaires from GBMSM in the U.K. and the Republic of Ireland. Ethical approval was granted by Glasgow Caledonian University (ref no: HLS/NCH/19/050). The survey was administered online via REDCap between June and July 2020 during the first COVID-19 lockdown. Adult GBMSM using gay-specific social media websites and/or apps (Grindr, Gaydar, Recon, Squirt, and Scruff) and/or mainstream social media (Facebook, Twitter, and Instagram) were invited to participate in their profile location, IP address or smartphone GPS co-ordinates were located in one of the five target countries (Scotland, Wales, Northern Ireland, England or the Republic of Ireland). Participants were also asked to circulate survey invites to relevant friends and family via email and text. In addition, our study collaborators sent invitations to their service users (HIV Scotland, Waverley Care SX, THT Scotland) to boost the Scottish sample since this country was the focus of our wider study. Similar to previous SMMASH surveys (e.g. McGarty et al., 2021), SMMASH-Pan examined mental, sexual, and physical health among GBMSM in the U.K. and Republic of Ireland, but also included questions on COVID-19 lockdown, social distancing and health service use. A copy of the questionnaire can be found on the SMMASH website https://www.smmash2020.org/surveys. The specific SMMASH-Pan measures examined herein are as follows;

*Demographics*: Age, sexual orientation (gay/bisexual or straight/other), education status (none/secondary or post-secondary), living situation (alone or with company) and ethnicity (white or person of colour).

*Mental and Physical Health*: Self-perceived health and mental health were assessed by asking participants ‘Currently, how is your (mental) health?’, with responses on a 5-point Likert-scale from ‘very good’ to ‘very poor’ and were also asked whether they had ever received a MH diagnosis by a doctor. Recent depression and anxiety were measured with the Patient Health Questionnaire-9 (PHQ-9 Kroenke, Spitzer, & Williams, 2001) and Generalized Anxiety Disorder-7 (GAD-7; Spitzer, Kroenke, Williams, & Löwe, 2006) questionnaires, respectively. PHQ-9 measures the frequency of various depression-related problems experienced in the last two weeks and has been validated for use in high income countries (Levis, Benedetti, Thombs, & DEPRESsion Screening Data (DEPRESSD) Collaboration, 2019). Responses are combined so participants could score between 0 (not affected by any of the issues at all) and 27 (affected by every issue nearly every day). Depression severity was then categorised as none/mild (score 0-9) or moderate/severe (score 10-27) depression. GAD-7 measures the frequency of various anxiety-related problems experienced in the last two weeks, with scores ranging from 0 (not affected by any issue at all) to 21 (affected by every issue nearly every day), validated for use in high income countries (Manea, Gilbody, & McMillan, 2015). Anxiety severity was then categorised as either none/mild anxiety (score 0-9) or moderate/severe (score 10-21) anxiety. Although these questionnaires are not necessarily diagnostic tools, people rated as experiencing moderate-/severe anxiety and/or depression would normally be considered by a clinician for pharmacological or counselling intervention (Manea et al., 2015).

*Remote MH help-seeking behaviour during the first COVID-19 lockdown*: Men were asked about their use of remote resources to seek MH support during lockdown as follows; ‘Have you accessed any of the following resources to help with your mental health since lockdown?’. Participants could select from ‘Websites’, ‘Apps’, ‘Phone Lines’, ‘None of the above’.

### Statistical analysis

An *a priori* power analysis was not conducted for the SMMASH-Pan survey in anticipation of achieving a large sample size and the exploratory nature of the project. Participants were asked not to complete this iteration of the survey if they had already done so, but duplicates were not screened for. Fraudulent and partial entries were screened and deleted. Given the large sample size of the study (*n* = 1801), outliers were included in the analysis. Data were analysed using SPSS version 24. Given the lack of existing evidence on factors predicting help seeking among GBMSM an exploratory statistical approach (backward binary logistic regression) was chosen over a theory-based (hierarchical binary logistic regression) statistical analysis to address RQ1. The backward binary logistic regression analysis calculated estimated odds ratios (ORs) and 95% confidence intervals (CIs) to explore factors associated with MH help-seeking behaviour during the first COVID-19 lockdown in GBMSM in the U.K. and Republic of Ireland. The final model contained all variables significant at the bivariate level (*P* < 0.05) to assess which remained statistically significant.

### Qualitative study

One-to-one phone and online interviews were conducted with 18 GBMSM living in Scotland between July and September 2020 (funding constraints meant interviews were only conducted with participants in Scotland). Ethical approval was obtained from Glasgow Caledonian University: ref no: HLS/NCH/19/054. Survey participants were asked to check a box if they would potentially take part in an interview to examine their experiences of lockdown and social distancing. To address self-selected bias of participants with MH issues being more likely to participate in those interviews, purposive sampling was used to identify interviewees from all potential participants, in three MH experience groups;

1. men who reported poor MH and had used MH online resources during lockdown,
2. men who reported poor MH and had not used MH online resources during lockdown,
3. men who reported good MH but nevertheless had used MH online resources during lockdown.

Maximum variation sampling (see Palinkas et al., 2015) was then used to obtain a diverse range of individuals by stratifying and recruiting potential participants across different gender, sexual identity, ethnicity, employment and living situation changes (experienced during the first COVID-19 lockdown), attempting to recruit equal numbers across the three MH experience groups.

An interview topic guide was used to facilitate discussion regarding men’s experiences of MH and MH help seeking during the first COVID-19 lockdown. Interviews were independently conducted by four authors of the paper (JF, PF, RM, DS). Interviewers were all white, cis-gendered and included both gay/straight and female/male researchers. DS led the analysis with support from PF.

### Qualitative data analysis

The 18 interviews were transcribed verbatim, anonymised and imported into NVIVO 12 for analysis. Deductive thematic analysis (Braun & Clarke, 2013) was employed to address RQ2, to identify the self-perceived barriers and facilitators to GBMSM’s MH help-seeking behaviour during the first COVID-19 lockdown in Scotland.

### Behaviour change wheel approach

To address RQ3, the barriers and facilitators to MH help-seeking were further analysed using the Behaviour Change Wheel (BCW) approach (Michie, Atkins, & West, 2014) by DS and verified by PF. The BCW was developed to provide a comprehensive, evidence-based, and theoretically informed guide to behaviour change. The BCW brings together 19 frameworks of behaviour change, each of which places greater emphasis on different drivers of behaviour. For example, some of these frameworks assume that behaviour is driven by beliefs and perceptions, while others highlight the role of the social environment in behaviour. By synthesising the core drivers of behaviour, the BCW adopts a holistic perspective, providing a model broad enough to be applied to any behaviour and setting (Michie et al., 2014).

Barriers and facilitators were conceptualised in behavioural terms, checking they relate to the prevention or facilitation of MH help-seeking by GBMSM. Then each of the identified barriers and facilitators were mapped to the Capability, Opportunity, Motivation - Behaviour (COM-B) model components (West & Michie, 2020) and corresponding Theoretical Domains Framework (TDF) domains (Atkins et al., 2017; Cane, O’Connor, & Michie, 2012). Next, the corresponding intervention functions were systematically selected to align with the selected TDF domains. Finally, we considered which Behaviour Change Techniques (BCTs) (Michie et al., 2013) could best operationalise the intervention functions, then generated draft recommendations which sought to systematically reduce the barriers we had identified and build on any facilitatory factors to enable GBMSM-facing organisations, services and online resources to meet unmet needs and enhance GBMSM’s MH help-seeking behaviours.

### Synthesis of findings to generate service provider and research recommendations

Following independent quantitative and qualitative data analysis, the key findings of each study were combined in a single matrix, to generate recommendations for clinical practice, community-based organisations, and future research. These initial recommendations were further synthesised by DS, PF, JF, and RMc (see Online Appendix, Table A3). Discussion enabled these authors to reach consensus and the final recommendations were further categorised as short, medium, or long-term (Table A3). For ease of reading, the final recommendations are presented within the discussion section of this paper.

## Findings

### Quantitative findings

RQ1: What are the key sociodemographic and psychological factors predicting MH help-seeking among GBMSM during the first COVID-19 lockdown in UK and Republic of Ireland?

#### Demographic characteristics

1368 of 1801 the SMMASH-Pan survey participants completed the MH resources questions and are included in the analysis. Demographic characteristics are shown in Table 1. The average age of participants was M = 41.5 years (SD = 13.5) with the largest sub-group aged 46+ (40.1%, *n* = 549). Almost all men identified themselves as gay or bisexual (98.5%, *n *= 1348). Most participants were living in Scotland (47.5%, *n* = 650) followed by England (21.0%, *n* = 287) and the Republic of Ireland (19.2%, *n* = 262), with fewer from Wales (7.5%, *n* = 103) and Northern Ireland (4.6%, *n* = 63). The vast majority self-identified as white (97%, *n* = 1327), most (88.1%, *n* = 1205) reported post-secondary school education and over one-third (37.7%, *n* = 516) were living alone at the time of participation. Overall, 38.3% (*n* = 520) reported ever receiving a MH diagnosis from a doctor, 35.9% (*n* = 470) were assessed as experiencing moderate to severe depression and 23.9% (*n* = 309) moderate to severe anxiety in the past 2 weeks. Although relatively few (8%, *n* = 109) men said that their physical health was poor/very poor, over one quarter (26.5%, *n* = 353) reported poor or very poor MH at the time of participating in the survey. Correspondingly, 21.7% (*n* = 297) of men reported using remote MH resources during lockdown.

**Table 1.** Demographic characteristics for all SMMASH-Pan Participants.

Demographic Variable (N total)	*N* (%)
Age (*n* = 1368)	
16–25 years	175 (12.8)
26–35 years	345 (25.2)
36–45 years	299 (21.9)
46 + years	549 (40.1)
Sexual Orientation (*n* = 1368)	
Gay	1170 (85.5)
Bisexual	178 (13.0)
Straight/Other	20 (1.5)
Country (*n* = 1368)	
Scotland	650 (47.5)
Wales	103 (7.5)
Northern Ireland	63 (4.6)
RoI	262 (19.2)
England	287 (21.0)
Other	3 (0.2)
Ethnicity (*n* = 1368)	
White	1327 (97.0)
People of colour	41 (3.0)
Education (*n* = 1368)	
None/ secondary	163 (11.9)
Post-secondary	1205 (88.1)
Living situation (*n* = 1368)	
Alone	516 (37.7)
With company	852 (62.3)
Remote MH Resources (*n* = 1368)	
Yes	297 (21.7)
No	1071 (78.3)
MH Diagnosis (*n* = 1358)	
No	838 (61.7)
Yes	520 (38.3)
Recent Depression (*n* = 1309)	
None/mild	839 (64.1)
Moderate/severe	470 (35.9)
Recent Anxiety (*n* = 1292)	
None/mild	983 (76.1)
Moderate/severe	309 (23.9)
Self-perceived Physical Health (*n* = 1358)	
Good/Very Good	1249 (92.0)
Poor/Very Poor	109 (8.0)
Self-perceived MH (*n* = 1354)	
Good/Very Good	991 (73.2)
Poor/Very Poor	363 (26.8)
Recent anxiety/depression or self-perceived poor MH combined (*n* = 1298)	
No	715 (55.1)
Yes	583 (44.9)
Accessed remote MH resources for men who report recent depression/anxiety or self-perceived poor MH (*n* = 583)	
No	400 (68.6)
Yes	183 (31.4)

Cumulatively 44.9% (*n* = 583) of participants reported either recent moderate/severe anxiety or depression or self-reported poor MH during the first COVID-19 lockdown. Amongst these men, just under one-third (31.4%, *n* = 183) had used remote MH resources since the start of the COVID-19 pandemic.

#### Predictors of remote MH help-seeking during the first COVID-19 lockdown

Table 2 presents the results of the backwards binary logistic regression analysis to predict remote MH help-seeking behaviour among GBMSM during lockdown. Variables entered into the constant model were age, education, self-perceived MH, ethnicity, self-perceived health, PHQ-9 depression, GAD-7 anxiety, lifetime MH diagnosis, living situation and sexual orientation. As Table 2 shows, the predictive independent variables included in the final model, that was set in 9 steps, were MH diagnosis and anxiety. This means that men suffering from moderate/severe anxiety and those having received a MH diagnosis in the past were more likely to report seeking help for MH issues since the start of the COVID-19 lockdown. None of the remaining variables were significant predictors of MH help-seeking in the final model. The internal validity of the model was good (Hosmer–Lemeshow = 0.26, *p* > 0.05, confirming goodness of fit).

**Table 2.** Constant only & final logistic regression model – coefficients of the model predicting MH help-seeking behaviour among GBMSM in the SMMASH-PAN study.

				95% CI for Odds Ratio	
Predictors	B	SE	Lower	Odds	Upper
CONSTANT MODEL					
Age	0.10	0.06	0.99	1.01	1.02
Post-secondary education (no)	0.19	0.23	0.77	1.21	1.90
MH (good)	0.17	0.20	0.80	1.18	1.74
Ethnicity (white)	0.11	0.41	0.50	1.12	2.47
Physical health (good)	−0.40	0.26	0.58	0.96	1.58
Recent Depression (none/mild)	0.11	0.19	0.76	1.12	1.64
Recent Anxiety (none/mild)	0.74***	0.20	1.41	2.09	3.11
MH diagnosis (no)	0.45**	0.15	1.16	1.56	2.10
Living situation (alone)	−0.18	0.16	0.62	0.84	1.14
Sex orientation (gay/bi)	−0.61	0.66	0.15	0.54	1.97
FINAL MODEL					
GAD (none/mild)	0.91***	0.16	1.83	2.48	3.36
MH diagnosis (no)	0.49**	0.15	1.22	1.62	2.17

***p < .01, **p < .05.

### Qualitative findings

RQ2: What are the barriers and facilitators to MH help-seeking among GBMSM in Scotland?

When discussing barriers and facilitators to MH help-seeking, qualitative interview participants distinguished between accessing online resources (specifically, websites and apps) and ‘GBMSM-facing’ organisations. Therefore, we use this distinction to structure our analysis as shown below.

#### Barriers and facilitators of MH help-seeking from GBMSM-facing organisations during the first COVID-19 lockdown

Multiple barriers and facilitators to men’s MH help-seeking from GBMSM-facing organisations were identified (see Table 3). Participants’ lack of knowledge about GBMSM-facing organisations and their lack of confidence to seek help from such organisations were described as major barriers impacting their help-seeking behaviour across our data. For example, men’s lack of knowledge around GBMSM-facing organisations is summarised below:

In terms of mental health and gay men, I had no idea that there were specific charities for that. I always thought, well, I’m gay, I have sex with men, if I did have a mental health issue and I wanted to speak to somebody about it I would just phone like a Samaritans or whatever, you know. It didn’t occur to me that there are specific charities that deal with gay men with those issues. I didn’t even know they existed, if I’m honest.

Similarly, some interviewees said they were discouraged from seeking help from GBMSM-facing organisations because they understood these organisations mainly focus upon sexual rather than MH issues.

**Table 3.** Key barriers & facilitators to GBMSM’s MH help-seeking from GBMSM-facing Organisations & Online Resources.

MH help-seeking – GBMSM-facing organisations	
Barriers	Facilitators
Lack of knowledge around GBMSM-facing organisations Lack of confidence to search for GBMSM-facing organisations Focus on sexual health Counselling perceived as lacking benefit Lack of motivation to seek help	Inclusivity of trans (including non-binary) men Socialisation chances with other gay men Counsellors with empathy Same sex identity counsellors/ freedom to select counsellor with diverse race characteristics or based on men’s individual needs Safe, inclusive, & comfortable environment Services discreetness
*MH help-seeking – online resources*	
Barriers	Facilitators
Lack of person-centred approach Attitudes towards self-resourcefulness Lack of perceived benefit Looking for ‘quick fix’ not ‘long term investment’ in MH	Referral by a trusted person Clear, concise, & discreet content and function Provision of individualised care Positive effect on MH

As a gay man, I don’t really feel all the sexual health obsession helps me as a gay man. It would help a lot more to be given more mental health support that goes beyond just handing out free condoms (at a gay) festival. I don’t want that. I want to be able to feel that I can actually have that conversation with somebody and express myself in a way that makes sense.

Other barriers to MH help-seeking included not knowing the benefits of MH support and not having the motivation/intention to seek help:

So, I think there probably are barriers to accessing (counselling) but I think some of those barriers are like internal in terms of not recognising the benefit that therapy might have.

I know there would be nothing bad about asking for help, it’s just one of those things like I don’t want to do it.

In addition, participants described a range of facilitators that would enable them to seek MH support from GBMSM-facing organisations (see Table 3). Endorsing a more inclusive approach towards trans (including non-binary) people and offering non-sexualised socialisation opportunities (e.g. outwith gay bars or commercial sex venues) for GBMSM were described by many interviewees as important factors enabling men’s MH help-seeking behaviours. Moreover, participants described the importance of GBMSM-facing organisations employing a person-centered counselling approach; many interviewees said they would happily seek help for MH issues from GBMSM-facing organisations if their counsellors clearly endorsed an empathic approach towards GBMSM or from counsellors with the same sexual identity as themselves. In addition, some interviewees suggested that having the freedom to select counsellors with diverse racial characteristics or based on their individual needs would motivate them to seek MH support from GBMSM-facing organisations.

So, you put all this effort into (a counsellor) who you know who is heterosexual, has different values than you do, and a different reasoning. You tend to hold back, you know, whereas (with) a gay man, or a … probably a gay man, I would be much more relaxed and able to talk to him in a way that it is easier for me to talk to him without having these other thoughts of … well, ‘I said that’ and then, if they are, you know, straight, then they would think that’s awful. They may not necessarily think that, but this is what I think they think.

Finally, some interviewees said feeling listened to and receiving support within a safe, inclusive, and discreet environment would also motivate some GBMSM to seek GBMSM-facing organisations’ help around MH issues. In the example below, the organisation’s acceptance of disability, as well as sexual identity, was important.

I go along to those (GBMSM-facing organisations) and feel like I’m understood quite well there because it’s a space where I can be (accepted) as a disabled person and as a queer person and that really feels nice to have that.

### Barriers and facilitators to MH help-seeking using online resources during the first COVID-19 lockdown

Our analysis identified three key barriers hindering men’s MH help-seeking using online resources; men distinguished between apps and websites, so we use this definition herein (see Table 3). GBMSM described (1) the lack of a tailored and person-centered approach employed by MH apps, (2) their perceptions of the likely low efficacy of online resources and (3) their belief that they are resourceful in themselves, as described below.

I wasn’t feeling the benefits (of a mental health app), enough, quick enough, so I don't know, I probably just gave up with that. So yeah, I wouldn’t really say that helped, but I did try it to begin with.

I have an app that I sometimes use on, I forget what it’s called, it’s like a mindfulness app. But I get sort of frustrated with these things when it tells you how to think and breathe. I think, ‘Oh I’d much rather go for a walk actually’ and think myself. So I guess I am sort of resourceful myself in some ways’.

The first extract also highlights how men expected MH supports, particularly apps, to provide a ‘quick fix’ to boost their MH, rather than requiring a longer-term investment to enhance their resilience, which led to rapid disengagement. On the other hand, our interviewees identified several facilitators that could boost their engagement with online MH resources (see Table 3). Some men said that referral to these apps by a trusted person would motivate them to seek help for MH issues through online resources:

Maybe if, you know, at (Hospital) or (MH organisation) someone I was seeing, some therapist, introduced me to an app and told me about it, I might have more interest.

Some interviewees also said that they would more easily engage with MH online resources if the functionality and aesthetics of these resources improved. For example, some men suggested that a more discreet, concise, and clear content and function would increase GBMSM’s engagement with online resources.

I was really distressed and I was looking for places, if … you went onto their site and there was bullet-point, bullet-point, bullet-point, that could then maybe help … does that make sense? ‘That’s the course of action I want to go down’ …  If there was something there to go, ‘bang, bang, bang, (That’s) what to do’. And then also you’ve got to be discreet, … , they don’t want it in their face, they just need somewhere to look (for) help.

Finally, individually tailored content was mentioned as an important facilitator to MH help-seeking. Similarly, a perceived beneficial effect upon men’s MH was reported as important factors to increase men’s help-seeking behaviour for online resources, as illustrated below.

I think sometimes particularly going to sleep, like I listen to the sleep cast function (of the app) on that just … to distract my conscious mind as I'm going to sleep. And I find that can be quite helpful to listen to something soothing as I drift off.

### Behaviour Change Wheel analysis

RQ3: What are the preliminary recommendations to enable services to enhance MH help-seeking among GBMSM?

Our analysis of the key barriers and facilitators to GBMSM’s MH help-seeking separately addresses (1) online resources and (2) GBMSM-facing organisations during the first COVID-19 lockdown. Tables A1 & A2 (in the online appendix) show our BCW analysis in detail.

#### Behavioural Domain: ‘GBMSM seek help for MH issues using online resources during the first COVID-19 lockdown

Key barriers and facilitators to MH help-seeking using online resources were linked to either reflective motivation (*n* = 3 barriers/facilitators) or physical opportunity (*n* = 3) within the COM-B model (see Table A1, in online appendix), meaning they were related to GBMSM’s motivation to perform the behaviour or the physical environment where the behaviour occurred, respectively. Next, the TDF was used to expand the COM-B components, acknowledging the explicit links between COM-B and the TDF; five of the 14 TDF domains were identified as being particularly important in relation to the barriers and facilitators to online help-seeking for MH. The majority of barriers and facilitators were theorised as relating to ‘Environmental context and resources’ (*n* = 3) and the ‘Beliefs about consequences’ (*n* = 2) domains. Corresponding ‘functions’ were then selected based on their resonance with the relevant TDF domains. In particular, four different intervention functions (Environmental restructuring (*n* = 3), Enablement (*n* = 3), Education (*n* = 3), Persuasion (*n* = 3)) were identified as likely to be useful in enhancing GBMSM’s MH help-seeking behaviour using online resources.

The penultimate step was to select BCTs that operationalised these intervention functions. BCTs selected to address the barriers and facilitators to MH help-seeking behaviour included ‘restructuring the physical environment’, ‘information about social and environmental consequences’, ‘information about emotional consequences’, ‘credible source’, ‘social support (unspecified)’, ‘framing/reframing’, ‘identity associated with changed behaviour’, and ‘incompatible beliefs’. Overall, these BCTs related to the broader BCT grouping ‘antecedents’ (*n* = 3), ‘natural consequences’ (*n* = 3), ‘associations’ (*n* = 3), ‘social support’ (*n* = 1), and ‘identity’ (*n* = 1).

Finally, by bringing together these BCTs, theoretically driven and systematic recommendations were generated that are expected to increase GBMSM’s help-seeking behaviour with the use of online resources (see final column, Table A1, in online appendix).

#### Behavioural Domain: ‘GBMSM seek help for MH issues from GBMSM-facing organisations during the first COVID-19 lockdown’

This same process was used to generate theoretically driven and evidence-based recommendations likely to enhance GBMSM’s MH help-seeking from GBMSM-facing organisations. Barriers and facilitators were mapped to the COM-B model as follows: reflective motivation (*n* = 7), followed by physical opportunity (*n* = 3), social opportunity (*n* = 2), and psychological capability (*n* = 2) (see Table A2, in online appendix). Using the TDF, these barriers and facilitators were theorised as relating to environmental context and resources (*n* = 7); professional/social role and identity (*n* = 4); cognitive and interpersonal skills (*n* = 1); social influences (*n* = 2); knowledge (*n* = 1); beliefs about capabilities (*n* = 1); intentions (*n* = 1); and beliefs about consequences (*n* = 1). Drawing on this analysis, the intervention functions seen as more likely to be effective in bringing about enhanced help-seeking behaviour with GBMSM-facing organisations were persuasion (*n* = 6), education (*n* = 5), environmental restructuring (*n* = 5), enablement (*n* = 2), modelling (*n* = 2), and training (*n* = 1).

Several BCTs were then identified based upon the selected intervention functions; ‘information about other’s approval’, ‘identification of self as role mode’, ‘restructuring the social environment’, ‘information about social and environmental consequences’, ‘demonstration of a behaviour’, ‘instruction on how to perform a behaviour’, ‘feedback on the behaviour’, ‘framing/reframing’, ‘information about emotional consequences’, ‘prompts/cues’, ‘focus on past success’, ‘imaginary reward’, ‘identity associated with changed behaviour’, and ‘incompatible beliefs’. These recommended BCTs related to the broader BCT groupings of ‘comparison of behaviour’ (*n* = 8), ‘natural consequences’ (*n* = 7), ‘identity’ (*n* = 5), ‘antecedents’ (*n* = 5), ‘shaping knowledge’ (*n* = 1), ‘feedback and monitoring’ (*n* = 1), ‘associations’ (*n* = 1), ‘self-belief’ (*n* = 1), ‘covert learning’ (*n* = 1).

Finally, the last column of Table A2 (see online appendix) illustrates the end product of our analyses and details the way behaviour change techniques could be operationalised to boost GBMSM’s MH help-seeking behaviour and enable GBMSM-facing organisations to address unmet needs both during the COVID-19 pandemic and, crucially, afterwards.

## Discussion

This is the first sequential mixed-methods study worldwide to examine MH help-seeking among GBMSM during the COVID-19 pandemic. For the first time, we have explored factors affecting GBMSM’s MH help-seeking behaviours during the pandemic in the U.K. and Republic of Ireland and identified barriers and facilitators to MH help-seeking among GBMSM in Scotland. By applying the BCW approach (Michie et al., 2014) we also performed a comprehensive analysis of the factors affecting MH help-seeking behaviours and systematically identified evidence and theory-based preliminary recommendations aimed at enhancing GBMSM’s MH help-seeking behaviours. The aim of this discussion is to examine and synthesise the key findings to generate short, medium, and long-term recommendations for clinical practice, NHS, GBMSM-facing and MH services, online resources, and future research (see Table 4).

**Table 4.** Short, medium & long-term recommendations to improve MH help-seeking among GBMSM in the U.K..

SHORT TERM RECOMMENDATIONS
**Mass & Social Media** – Advertise existing resources to improve MH (e.g. apps, websites, helplines, counselling) for GBMSM. **Mass & Social Media** – Raise awareness of simple/useful MH promotion techniques (e.g. exercise, social interaction, reduce alcohol/drugs, mindfulness etc.) for GBMSM. **GBMSM & MH services** to raise awareness of success of existing resources to help improve MH (for GBMSM). Since those with longstanding MH issues already know how to seek help, **GBMSM and MH** services should focus on those with new MH problems**.** Ensure **GBMSM-facing organisations** advertise MH services they offer within wider promotion of their services. **GBMSM & MH services** to raise awareness of existing MH resources. Working with national, regional, and local stakeholders to consider the cocreation of a GBMSM population targeted mass and social media intervention to reach diverse GBMSM. Ensure ‘branding’ is inclusive and visibly welcome to all – celebrate diversity of services users and, where possible, staff.
MEDIUM TERM RECOMMENDATIONS
Develop GBMSM-facing/tailored resources to help men with poor MH. Frame MH help seeking as normative for GBMSM and positively endorsed. Consider innovative ways by which these men already seeking help for MH can share their experience to enable others to also do so. Promote MH maintenance as a long-term commitment (i.e. Ruby Wax ‘deposit in saving bank’) not a ‘quick fix’ (i.e. one session of meditation unlikely to make you feel better). Communicate these issues via a webinar and linked short briefing in order to ensure consistency across the sector. Primary focus is to embed good practice within service delivery. Important to think of social and discrete spaces for both **physical and digital services**. Contemporary **research** needed to further understand what factors affect the MH of GBMSM (e.g. impact of minority stress, homophobia, disproportionate substance us) and, critically, how they interact with other health inequalities to produce syndemic ill-health among this group. **Mass & social media** advertise testimonials of use and demonstration of how to access services. Work with **GBMSM** and **lobby boards** for MH inclusion. **Peers, partners, friends & family** can play a role in enhancing GBMSM confidence and self-efficacy in help-seeking for (online) MH resources – ‘help a friend’. Run webinar so **GBMSM MH ambassadors** describe how they successfully searched for, and used, services for the first time. **GBMSM Ambassadors** can operate at the organisational (e.g. THT, Waverley Care-SX), community (e.g. BearScots, FrontRunners, gay/straight alliance in educational settings), professional (GPs, STI clinician), celebrity (Lady Gaga) and individual (e.g. Jimmy MacDonald) level. **GBMSM MH Ambassadors** frame MH as an issue for GBMSM, endorse and promote (i) MH help-seeking, (ii) MH resources and organisations, (iii) high-quality MH apps, using personal testimonials. **GBMSM and MH organisations** coproduce MH materials with **GBMSM community** Connect **MH with SH** and service use more broadly (holistic health). **Research, MH and GBMSM organisations and communities** work collaboratively to identify those high-quality MH apps (already identified in systematic reviews) most suitable for GBMSM. **Research, GBMSM/MH Organisations and GBMSM community** work together to design GBMSM-tailored MH resources (e.g. meditation programme) using existing modifiable apps (e.g. InsightTimer). **Local GBMSM services / community ambassadors** provide support in using the apps (LGBT MH app support group, training intervention). After quality appraisal, **GBMSM and MH services** advocate relevance and utility of specific GBMSM-appropriate MH resources. **MH services** should be proactive to reach GBMSM. **NHS/GBMSM-facing organisations** devise strategies to ensure digitally excluded GBMSM are included when services are delivered remotely. Rebranding of **GBMSM-facing organisations** to include MH issues. Moving away from GBMSM-facing organisations due to ending HIV. **GBMSM-facing services** develop and deliver GBMSM staff training package for generic MH and wider services. **GBMSM-facing organisations** endorse and promote those high-quality MH apps suitable to GBMSM, via outreach work, social & mass media. **GBMSM-facing organisations** should adapt outreach activities to promote MH and Wellbeing and ensure their MH services are promoted as widely as possible.
LONG TERM RECOMMENDATIONS
Create community-development initiatives, drawing on assets-based co-creation approach to enhance mental health. Advocate importance of MH awareness and maintenance for **all**. Advocate importance of MH awareness and maintenance for GBMSM population given their unique multiple vulnerabilities (syndemics). Collaborative research with **GBMSM communities, MH & GBMSM organisations and app designers** to coproduce MH app tailored to GBMSM. RCT to evaluate the efficacy of MH app tailored to GBMSM. Create new identity of MH maintainer (like exercising, 5-a-day etc.) for GBMSM as someone who actively engages in activities to maintain their mental health (e.g. exercise, meditation etc.). Work to ensure **generic MH organisations** dealing with specific issues (alcohol, drugs, abuse, eating disorders, MH etc.) are gay friendly. High-level work is needed to make sure that **commissioners** and service-level agreements achieve coherence and clarity around who delivers what and to whom. National level service provision (e.g. Scotland-wide, U.K.-wide) could enable celebration of diversity of service provider staff more realistically.

### Short, medium & long-term recommendations

Our short-term recommendations mainly focus upon raising GBMSM’s awareness **around** existing evidence-based resources that can improve their MH, increasing the visibility of MH services appropriate to GBMSM, such that diverse GBMSM and those with new MH problems are reached. To these ends, we recommend services’ branding is inclusive and visibly welcoming to all GBMSM by celebrating diversity of services users and, where possible, staff (see Table 4).

In the medium term (see Table 4) we recommend a holistic and collaborative approach between researchers, communities, GBMSM-facing organisations, and MH services is adopted to develop MH resources for GBMSM. This includes identifying and promoting existing high quality MH resources, especially materials and apps appropriate to GBMSM’s needs. GBMSM and MH ambassadors could fruitfully cooperate at different levels to promote and support (i) MH help-seeking, (ii) MH resources and organisations and (iii) high-quality evidence-based MH apps, using personal testimonials from community members. Significant others, such as peers and partners, could also play a role in enhancing GBMSM’s confidence and self-efficacy in MH help-seeking. Moreover, it is suggested that GBMSM-facing organisations endorse a more holistic rather than a sexual health focus by (i) rebranding their services to explicitly promote MH issues, (ii) developing and delivering a GBMSM staff training package for generic MH and wider services, (iii) promoting high-quality MH apps suitable to GBMSM, and (iv) adapting outreach activities to promote MH. GBMSM-facing organisations should also ensure their MH services are promoted as widely as possible and include digitally excluded GBMSM when services are delivered remotely. Future research should explore those factors affecting the MH of GBMSM and, critically, their interaction with other health inequalities which produce syndemic ill-health among GBMSM (McDaid et al., 2020). Social and discrete spaces should be developed for physical and digital services, whilst communicating MH issues via webinars and short briefings will ensure consistency across the sectors. It is proposed that future mass and social media campaigns advertise personal testimonials of MH service use that explain how to successfully access services, and GBMSM-tailored resources need to be developed that support GBMSM with poor MH. Finally, and perhaps most importantly, MH help-seeking among GBMSM should be framed as normative, while MH maintenance should be re-framed as a long-term commitment rather than a ‘quick fix’.

Our long-term recommendations advocate an inclusive MH approach be promoted across different organisational levels that emphasises the importance of MH awareness and maintenance for all. Raising awareness about GBMSM’s MH should also be prioritised across different services, given the unique multiple vulnerabilities (i.e. syndemics) of the GBMSM population. Based on our recommendations, researchers should collaborate with GBMSM communities, GBMSM-facing and MH organisations alongside app designers to both endorse an asset-based approach towards GBMSM’s MH and co-produce MH apps tailored to GBMSM’s needs. The effectiveness and efficacy of these newly designed MH apps should be further examined within RCT interventions. Boldly, we propose that GBMSM could be guided to re-frame their identity as ‘MH maintainers’, that is someone who actively engages in activities to maintain their MH. Finally, further work is needed to ensure that generic U.K.-based MH organisations are GBMSM friendly whilst, where possible, national-level services should celebrate of diversity of their staff to promote diversity and inclusivity (see Table 4).

### Importance and implications of the results

The results of the quantitative analysis suggest that men experiencing recent anxiety and those with a previous MH diagnosis were most likely to access MH resources during the COVID-19 lockdown, regardless of any socio-demographic variables. Thus, it is important to enhance access to resources for those men with recent depression and those experiencing new, undiagnosed mental health problems. The latter are particularly challenging to identify, but prioritising GBMSM as a vulnerable population alongside promoting the importance of mental health awareness and maintenance for all GBMSM would begin to address this issue. Our qualitative analysis identified a series of issues, across individual and contextual factors, that GBMSM said influenced their help-seeking behaviour from online MH resources and GBMSM-facing organisations. These factors must be acknowledged by the relevant organisations, in order to develop more tailored and inclusive approaches that reach a larger, more diverse group of GBMSM.

### Limitations and future research recommendations

The key strength of this study is its originality; this is the first study to examine barriers and facilitators to MH help-seeking among GBMSM globally during the COVID-19 pandemic. The mixed-methods design strengthens research rigour, compensating the shortcomings of each individual methodology. In addition, employing the BCW approach enabled us to draw upon a wealth of prior theoretical work to assist with the systematic development of recommendations to enhance GBMSM’s engagement with MH services (Michie et al., 2014). However, the inherent limitations of cross-sectional research, our hybrid online/offline sampling frame, as well as the sample demographic characteristics, limited generalisability of these data; most participants were highly educated, self-identified as gay and our 5 countries were unequally represented. Moreover, another limitation concerns the skewed age of our sample and the subsequent utilisation of remote, especially *online*, resources that may differ by age. Similarly, recruiting interview participants only in Scotland, albeit a diverse, theory-driven sample, potentially limited the transferability of these qualitative findings. Although the use of social media resources to recruit participants yielded rapid results and provided anonymity in questionnaire and interview responding, it could also have biased the sample towards individuals utilising technology for support already. Due to funding limitations, this study did not include follow-up data collection during subsequent U.K. lockdowns. However, it is likely that MH and gay facing organisations implemented a number of these recommendations over the course of the pandemic. Similarly, as the pandemic continued, GBMSM may have shifted their help-seeking behaviours to utilise more online resources, and this is not reflected in the findings of this study. In addition, selection of intervention functions and BCTs is inevitably subject to some interpretation by the authors. This could be addressed if, for example, follow-up interviews with GBMSM were conducted, where men were asked to gauge which BCTs were most likely to be successful in improving their MH help-seeking behaviour. Finally, although remote help-seeking examined herein included the use of MH apps and websites, online support groups and telehealth, that are potentially other important ways to meet GBMSM's mental health needs, were not examined.

Finally, we have presented a range of systematic and theoretically informed recommendations to increase GBMSM’s engagement with MH resources, including GBMSM-facing organisations and online resources, developed herein with the use of the BCW approach. As such, they are ready to be implemented into practice. Our next step is to use these recommendations to develop a focused intervention that will aim to improve the MH of GBMSM in Scotland during and beyond COVID-19. We will review the feasibility and appropriateness of these recommendations against the APEASE criteria (Affordability, Practicability, Effectiveness/Cost-Effectiveness, Acceptability, Safety and Equity) with the help of expert key stakeholders and GBMSM (Michie et al., 2014), then employ follow-up qualitative interviews with GBMSM, GBMSM-facing, and MH service providers to gauge BCT feasibility and potential efficacy, in order to further enhance the rigour of these recommendations.

## Ethical approval

Nursing Department Research Ethics Committee, Glasgow Caledonian University (HLS/NCH/19/050; HLS/NCH/19/054).

## Supplementary Material

Supplemental Material

## Note

1 Services for whom a key remit is to work with GBMSM or who identify GBMSM as one of their key client groups.

## References

[CIT0001] Atkins, L., Francis, J., Islam, R., O’Connor, D., Patey, A., Ivers, N., … Grimshaw, J. M. (2017). A guide to using the theoretical domains framework of behaviour change to investigate implementation problems. *Implementation Science*, *12*(1), 77. 28637486 10.1186/s13012-017-0605-9 PMC5480145

[CIT0002] Bavinton, B. R., Chan, C., Hammoud, M. A., Maher, L., Haire, B., Degenhardt, L., … Prestage, G. P. (2022). Increase in depression and anxiety among Australian Gay and bisexual men during COVID-19 restrictions: Findings from a prospective online cohort study. *Archives of Sexual Behavior*, *51*, 355–364. doi:10.1007/s10508-021-02276-2 35039984 PMC8763302

[CIT0003] Bradford, S., & Rickwood, D. (2014). Adolescent's preferred modes of delivery for mental health services. *Child and Adolescent Mental Health*, *19*(1), 39–45. 32878356 10.1111/camh.12002

[CIT0004] Braun, V., & Clarke, V. (2013). *Successful qualitative research: A practical guide for beginners sage*. London: Sage Publications Ltd.

[CIT0005] Cane, J., O’Connor, D., & Michie, S. (2012). Validation of the theoretical domains framework for use in behaviour change and implementation research. *Implementation Science*, *7*(1), 37. doi:10.1186/1748-5908-7-37 22530986 PMC3483008

[CIT0006] Cheesmond, N. E., Davies, K., & Inder, K. J. (2019). Exploring the role of rurality and rural identity in mental health help-seeking behavior: A systematic qualitative review. *Journal of Rural Mental Health*, *43*(1), 45–59. doi:10.1037/rmh0000109

[CIT0007] Coleman, S. J., Stevelink, S. A. M., Hatch, S. L., Denny, J. A., & Greenberg, N. (2017). Stigma-related barriers and facilitators to help seeking for mental health issues in the armed forces: A systematic review and thematic synthesis of qualitative literature. *Psychological Medicine*, *47*(11), 1880–1892. 28290262 10.1017/S0033291717000356

[CIT0008] Collin, P. J., Metcalf, A. T., Stephens-Reicher, J. C., Blanchard, M. E., Herrman, H. E., Rahilly, K., & Burns, J. M. (2011). Reachout. Com: The role of an online service for promoting help-seeking in young people. *Advances in Mental Health*, *10*(1), 39–51.

[CIT0009] Dean, E. (2017). Scotland's new mental health strategy. *Mental Health Practice (2014)*, *20*(9), 10.

[CIT0010] Divin, N., Harper, P., Curran, E., et al. (2018). Help-seeking measures and their use in adolescents: A systematic review. *Adolescent Resarch Review*, *3*, 113–122. doi:10.1007/s40894-017-0078-8

[CIT0011] Firth, J., Torous, J., Nicholas, J., Carney, R., Pratap, A., Rosenbaum, S., & Sarris, J. (2017b). The efficacy of smartphone-based mental health interventions for depressive symptoms: A meta-analysis of randomized controlled trials. *World Psychiatry*, *16*, 287–298. doi:10.1002/wps.20472 28941113 PMC5608852

[CIT0012] Firth, J., Torous, J., Nicholas, J., Carney, R., Rosenbaum, S., & Sarris, J. (2017a). Can smartphone mental health interventions reduce symptoms of anxiety? A meta-analysis of randomized controlled trials. *Journal of Affective Disorders*, *218*, 15–22. doi:10.1016/j.jad.2017.04.046 28456072

[CIT0013] Frankis, J., Strongylou, D., & Kincaid, R. (2020). *How has Covid-19 social distancing amplified the mental health vulnerabilities of gay, bisexual and other men who have sex with men (GBM)? COV/GCU/20/10, Rapid Research in Covid-19 programme (RARC-19)*. https://www.cso.scot.nhs.uk/wp-content/uploads/COVGCU2010-1.pdf

[CIT0014] Gál, É, Ștefan, S., & Cristea, I. A. (2021). The efficacy of mindfulness meditation apps in enhancing users’ well-being and mental health related outcomes: A meta-analysis of randomized controlled trials. *Journal of Affective Disorders*, *279*, 131–142. 33049431 10.1016/j.jad.2020.09.134

[CIT0015] Galdas, P. M., Cheater, F., & Marshall, P. (2005). Men and health help-seeking behaviour: Literature review. *Journal of Advanced Nursing*, *49*(6), 616–623. 15737222 10.1111/j.1365-2648.2004.03331.x

[CIT0016] Gulliver, A., Griffiths, K. M., & Christensen, H. (2010). Perceived barriers and facilitators to mental health help-seeking in young people: A systematic review. *BMC Psychiatry*, *10*(1), 1–9. 21192795 10.1186/1471-244X-10-113 PMC3022639

[CIT0017] Hoffberg, A. S., Stearns-Yoder, K. A., & Brenner, L. A. (2020). The effectiveness of crisis line services: A systematic review. *Frontiers in Public Health*, *7*, 399. 32010655 10.3389/fpubh.2019.00399 PMC6978712

[CIT0018] Holloway, I. W., Garner, A., Tan, D., Ochoa, A. M., Santos, G. M., & Howell, S. (2021). Associations between physical distancing and mental health, sexual health and technology use among gay, bisexual and other men who have sex with men during the COVID-19 pandemic. *Journal of Homosexuality*, *68*(4), 692–708. 33528316 10.1080/00918369.2020.1868191

[CIT0019] Kauer, S. D., Mangan, C., & Sanci, L. (2014). Do online mental health services improve help-seeking for young people? A systematic review. *Journal of Medical Internet Research*, *16*(3), e66. 24594922 10.2196/jmir.3103 PMC3961801

[CIT0020] Kroenke, K., Spitzer, R. L., & Williams, J. B. (2001). The PHQ-9: Validity of a brief depression severity measure. *Journal of General Internal Medicine*, *16*(9), 606–613. 11556941 10.1046/j.1525-1497.2001.016009606.x PMC1495268

[CIT0021] Levis, B., Benedetti, A., Thombs, B. D., & DEPRESsion Screening Data (DEPRESSD) Collaboration. (2019). Accuracy of patient health questionnaire-9 (PHQ-9) for screening to detect major depression: Individual participant data meta-analysis. *BMJ* (Clinical research ed.), *365*, l1476. doi:10.1136/bmj.l1476

[CIT0022] Manea, L., Gilbody, S., & McMillan, D. (2015). A diagnostic meta-analysis of the Patient Health Questionnaire-9 (PHQ-9) algorithm scoring method as a screen for depression. *General Hospital Psychiatry*, *37*(1), 67–75. 25439733 10.1016/j.genhosppsych.2014.09.009

[CIT0001a] McDaid, L., Flowers, P., Ferlatte, O., McAloney, K., Gilbert, M., & Frankis J. (2020) Informing theoretical development of salutogenic, asset-based health improvement to reduce syndemics among gay, bisexual and other men who have sex with men: Empirical evidence from secondary analysis of multi-national, online cross-sectional surveys. *Social Science and Medicine: Population Health*, *10*(4). doi:10.1016/j.ssmph.2019.100519 PMC6911981 31853476

[CIT0023] McDermott, E. (2015). Asking for help online: Lesbian, gay, bisexual and trans youth, self-harm and articulating the ‘failed’self. *Health*, *19*(6), 561–577. 25413341 10.1177/1363459314557967

[CIT0024] McGarty, A., McDaid, L., Flowers, P., Riddell, J., Pachankis, J., & Frankis, J. (2021). Mental health, potential minority stressors and resilience: Evidence from a cross-sectional survey of gay, bisexual and other men who have sex with men within the Celtic nations. *BMC Public Health*, *21*(1), 1–15. 34742262 10.1186/s12889-021-12030-x PMC8572060

[CIT0025] Michie, S., Atkins, L., & West, R. (2014). *The behaviour change wheel. A guide to designing interventions* (1st ed.). Great Britain: Silverback Publishing. p. 1003–1010

[CIT0026] Michie, S., Richardson, M., Johnston, M., Abraham, C., Francis, J., Hardeman, W., … Wood, C. E. (2013). The behavior change technique taxonomy (v1) of 93 hierarchically clustered techniques: Building an international consensus for the reporting of behavior change interventions. *Annals of Behavioral Medicine*, *46*(1), 81–95. 23512568 10.1007/s12160-013-9486-6

[CIT0027] Moore, S. E., Wierenga, K. L., Prince, D. M., Gillani, B., & Mintz, L. J. (2021). Disproportionate impact of the COVID-19 pandemic on perceived social support, mental health and somatic symptoms in sexual and gender minority populations. *Journal of Homosexuality*, *68*(4), 577–591. 33399504 10.1080/00918369.2020.1868184

[CIT0028] Murphy, M. P. (2020). COVID-19 and emergency eLearning: Consequences of the securitization of higher education for post-pandemic pedagogy. *Contemporary Security Policy*, *41*(3), 492–505.

[CIT0029] Palinkas, L. A., Horwitz, S. M., Green, C. A., Wisdom, J. P., Duan, N., & Hoagwood, K. (2015). Purposeful sampling for qualitative data collection and analysis in mixed method implementation research. *Administration and Policy in Mental Health and Mental Health Services Research*, *42*(5), 533–544. 24193818 10.1007/s10488-013-0528-y PMC4012002

[CIT0030] Peterson, Z. D., Vaughan, E. L., & Carver, D. N. (2020, October 5). Sexual identity and psychological reactions to COVID-19. *Traumatology*. Advance Online Publication, doi:10.1037/trm0000283

[CIT0031] Pierce, B. S., Perrin, P. B., Tyler, C. M., McKee, G. B., & Watson, J. D. (2021). The COVID-19 telepsychology revolution: A national study of pandemic-based changes in U.S. Mental health care delivery. *American Psychologist*, *76*(1), 14–25. doi:10.1037/amp0000722 32816503

[CIT0032] Planey, A. M., Smith, S. M., Moore, S., & Walker, T. D. (2019). Barriers and facilitators to mental health help-seeking among African American youth and their families: A systematic review study. *Children and Youth Services Review*, *101*, 190–200.

[CIT0033] Prestage, G., Hammoud, M., Jin, F., Degenhardt, L., Bourne, A., & Maher, L. (2018). Mental health, drug use and sexual risk behavior among gay and bisexual men. *International Journal of Drug Policy*, *55*, 169–179. 29429865 10.1016/j.drugpo.2018.01.020

[CIT0034] Pretorius, C., Chambers, D., & Coyle, D. (2019). Young people’s online help-seeking and mental health difficulties: Systematic narrative review. *Journal of Medical Internet Research*, *21*(11), e13873. 31742562 10.2196/13873 PMC6891826

[CIT0035] Rathbone, A. L., Clarry, L., & Prescott, J. (2017). Assessing the efficacy of mobile health apps using the basic principles of cognitive behavioral therapy: Systematic review. *Journal of Medical Internet Research*, *19*(11), e399. 29187342 10.2196/jmir.8598 PMC5727354

[CIT0036] Read, J., & Cain, A. (2013). A literature review and meta-analysis of drug company–funded mental health websites. *Acta Psychiatrica Scandinavica*, *128*(6), 422–433. 23662697 10.1111/acps.12146

[CIT0037] Reavley, N. J., & Jorm, A. F. (2011). The quality of mental disorder information websites: A review. *Patient Education and Counseling*, *85*(2), e16–e25. 21087837 10.1016/j.pec.2010.10.015

[CIT0038] Rickwood, D., Deane, F., Wilson, C., & Ciarrochi, J. (2005). Young people’s help-seeking for mental health problems. *Australian e-Journal for the Advancement of Mental Health*, *4*(3), 3–34.

[CIT0039] Rickwood, D., Thomas, K., Bradford, S., & The Sax Institute. (2012). *Help-seeking measures in mental health: A rapid review*. https://www.saxinstitute.org.au/wp-content/uploads/02_Help-seeking-measures-in-mental-health.pdf

[CIT0040] Rogers, M. A., Lemmen, K., Kramer, R., Mann, J., & Chopra, V. (2017). Internet-delivered health interventions that work: Systematic review of meta-analyses and evaluation of website availability. *Journal of Medical Internet Research*, *19*(3), e7111. 10.2196/jmir.7111 PMC5384996 28341617

[CIT0041] Spitzer, R. L., Kroenke, K., Williams, J. B., & Löwe, B. (2006). A brief measure for assessing generalized anxiety disorder: The GAD-7. *Archives of Internal Medicine*, *166*(10), 1092–1097. 16717171 10.1001/archinte.166.10.1092

[CIT0042] Suen, Y. T., Chan, R. C., & Wong, E. M. Y. (2020). Effects of general and sexual minority-specific COVID-19-related stressors on the mental health of lesbian, gay, and bisexual people in Hong Kong. *Psychiatry Research*, *292*, 113365. 32862107 10.1016/j.psychres.2020.113365 PMC7397990

[CIT0043] West, R., & Michie, S. (2020). *A brief introduction to the COM-B Model of behaviour and the PRIME theory of motivation* [v1]. *Qeios*.

[CIT0044] Wu, C. Y., & Lee, M. B. (2021). Suicidality, self-efficacy and mental health help-seeking in lesbian, gay, bisexual and transgender adults in Taiwan: A cross-sectional study. *Journal of Clinical Nursing*, *30*(15-16), 2270–2278. 33529443 10.1111/jocn.15680

[CIT0045] Xu, Z., Huang, F., Kösters, M., Staiger, T., Becker, T., Thornicroft, G., & Rüsch, N. (2018). Effectiveness of interventions to promote help-seeking for mental health problems: Systematic review and metaanalysis. *Psychological Medicine*, *48*, 2658–2667. doi:10.1017/S0033291718001265 29852885

[CIT0046] Zay, H., Tam, M. K., Au, C. L., Yeoh, S. Y., Tan, G., Lee, M. M., … V, V. (2021). Barriers and facilitators to professional mental health help-seeking behavior: Perspective of Malaysian LGBT individuals. *Journal of LGBTQ Issues in Counseling*, *15*(1), 38–58.

