# Supplementary material for: Understanding and responding to remote mental health help-seeking by gay, bisexual and other men who have sex with men (GBMSM) in the U.K. and Republic of Ireland: a mixed-method study conducted in the context of COVID-19
Source: Health Psychol Behav Med. 2022 Apr 5;10(1):357–78. doi: 10.1080/21642850.2022.2053687 (PMC8986177; doi:10.1080/21642850.2022.2053687)
Supplement: Supplemental Material [file RHPB_A_2053687_SM4291.docx]

**Online Appendix**

| **Table A1**. Barriers and Facilitators to GBMSM’s mental health (MH) help-seeking from online resources during the Covid-19 pandemic; analysed using the BCW approach or BCW analysis | | | | | |
| --- | --- | --- | --- | --- | --- |
| **Factor** | **Com-b** | **TDF** | **Intervention Function** | **BCTs** | **Recommendations** |
| **Facilitators** |  |  |  |  |  |
| GBMSM find it easy to seek help for MH issues from online resources **because these provide individually tailored care** | Physical opportunity | Environmental context and resources | Environmental restructuring | 12.1 Restructuring the physical environment | Advise designers to develop individually tailored MH resources. |
| GBMSM find it easy to seek help for MH issues from online resources **because they believe using these has a direct positive effect upon their MH** | Reflective motivation | Beliefs about consequences | Education  Persuasion | 5.3 Information about social and environmental consequences  5.6 Information about emotional consequences  9.1 Credible source | Inform GBMSM about the number and types of people who benefited from MH online resources via social media, local media, or health services.  Inform GBMSM about the beneficial effect of MH online resources upon GBMSM’s MH via social media, local media, or health services.    Through relevant media use culturally appropriate leaders (e.g. key opinion leaders) to emphasise the positive effect of using MH online resources upon GBMSM’s MH. |
| GBMSM find it easy to seek help for MH issues in online resources because **the online resources are** **discreet, concise, and clear** | Physical opportunity | Environmental  context and  resources | Environmental restructuring  Enablement | 12.1 Restructuring the physical environment | Advise designers to develop MH online resources which are discreet, succinct, and clear. |
| GBMSM find it easy to seek help for MH issues in online resources **because GBMSM are referred to these online resources by a trusted person** | Social opportunity | Social influences | Enablement  Modelling | 3.1 Social support (unspecified)  9.1 Credible source | Ask service providers to endorse seek MH support using MH online resources.    Provide *vox-pops* where GBMSM advise of their own positive use of MH online resources.  Through relevant media use culturally appropriate leaders (e.g. key opinion leaders) to endorse and explain the use and benefits of MH online resources. |
| **Barriers** |  |  |  |  |  |
| GBMSM find it hard to seek help for MH issues in online resources **because GBMSM believe themselves to be resourceful, and thus, they don’t need any MH resources** | Reflective motivation | Beliefs about capabilities  Social/  professional role and identity | Education  Persuasion  Enablement | 5.6 Information about emotional consequences  13.2 Framing/  reframing  13.5 Identity associated with changed behaviour  13.3 Incompatible beliefs | Through mass or social media, and/or health services, Inform GBMSM (with the use of brief texts or infographics) about the number and various types of people whose MH benefited from MH online resources.  In local media, hook-up apps, suggest that men might think of the online MH resources as a self-help tool (rather than as an independent source of help).  Ask the person to articulate their new identity as someone who engages with MH resources.  Draw attention to GBMSM’s rejection of MH online resources and their thoughts about the importance of improving their MH. |
| GBMSM find it hard to seek help for MH issues in online apps **because they don’t find that they have benefited from their past use of online resources** | Reflective motivation | Beliefs about consequences | Education  Persuasion | 5.3 Information about social and environmental consequences  5.6 Information about emotional consequences  9.1 Credible source | Through mass or social media, and/or health services, inform GBMSM about the number of people benefited from MH online resources.  Through mass or social media, and/or health services inform GBMSM about the beneficial effect of MH online resources upon GBMSM’s emotional health.  Through Social media, local media and/or health services use culturally appropriate leaders (e.g. key opinion leaders) to emphasise the positive effect of using MH online resources upon GBMSM’s MH. |
| GBMSM find it hard to seek help for MH issues in online resources **because these resources are not individualized/tailored to each person’s needs** | Physical opportunity | Environmental context and resources | Environmental restructuring | 12.1 Restructuring the physical environment | Work closely with apps/websites designers to consider developing individually tailored MH online resources. |

| **Table A2.** Barriers and Facilitators to GBMSM’s mental health (MH) help-seeking in GBMSM-facing organisations during the Covid-19 pandemic; the BCW approach | | | | | |
| --- | --- | --- | --- | --- | --- |
| **Factor** | **Com-b** | **TDF** | **Intervention Function** | **BCTs** | **Recommendations** |
| **Facilitators** |  |  |  |  |  |
| GBMSM find it easy to seek help for gay related MH issues in GBMSM-facing organisations because **GBMSM-facing organisations are inclusive of non-binary/trans** | Reflective motivation  Physical opportunity | Professional/social role and identity  (GBMSM-facing organisations)  Environmental context and resources | Persuasion  Education  Environmental restructuring | 6.3 Information about other’s approval  13.1 identification of self as role mode  12.2 Restructuring the social environment | Work closely with GBMSM-facing organisations to ensure they know the high value men place on services being inclusive of gender identity.  Work closely with GBMSM-facing organisations to ensure they understand that if they are clearly inclusive of diverse gender identities, they may be a good example for other GBMSM-facing organisations.  Ask GBMSM-facing organisations to develop programmes that target diverse gender identities. |
| GBMSM find it easy to seek help for gay related MH issues in GBMSM-facing organisations **because they receive counselling from same sex identity counsellors; race diverse counsellors; can select counsellors (sex identity) according to individual needs** | Reflective motivation  Physical opportunity | Professional/social role and identity  Environmental context and resources | Persuasion  Environmental restructuring | 6.3 Information about other’s approval  5.3 Information about social and environmental consequences  12.2 Restructuring the social environment | Work closely with GBMSM-facing organisations to ensure they know the high value men place on hiring counsellors who are diverse in relation to sexual, gender and ethnic identity.  Work closely with GBMSM-facing organisations to ensure they are aware of the large number of MSM seeking diverse counsellors.  Work closely with GBMSM-facing organisations to ensure they hire counsellors who highly diverse. |
| GBMSM find it easy to seek help for gay related MH issues in GBMSM-facing organisations **because they receive counselling from counsellors with empathy**  GBMSM find it easy to seek help for gay related MH issues in GBMSM-facing organisations **because they feel listened to & understood** | Psychological capability  Reflective motivation | Environmental context and resources  Cognitive and interpersonal Skills  Professional/social role and identity | Training  Education | 4.1 Instruction on how to perform a behaviour | Work with counsellors and enhance their professional competencies with regard to overt demonstrations of empathy. |
| GBMSM find it easy to seek help for gay related MH issues in GBMSM-facing organisations **because these services are discreet** | Physical opportunity | Environmental context and resources | Environmental restructuring | 12.1 Restructuring the physical environment | Work closely with GBMSM-facing organisations to develop discreet contact options for accessing services (e.g. smaller contact cards, private Zoom calls). |
| GBMSM find it easy to seek help for gay related MH issues in GBMSM-facing organisations **because they feel safe and comfortable** | Social opportunity | Environmental context and resources | Environmental restructuring | 12.1 Restructuring the physical environment | Work closely with GBMSM-facing organisations to make environmental changes (decoration/ place changes) to increase the sense of feeling safe and comfortable. |
| GBMSM find it easy to seek help for gay related MH issues in GBMSM-facing organisations **because they offer opportunities for socialisation with other gay men** | Social opportunity | Environmental context and resources  Social influences | Environmental restructuring  Enablement | 12.2 restructuring the social environment  13.2  Framing/  Re-framing | Work closely with GBMSM-facing organisations to develop independent peer meetings where GBMSM will have the chance to talk and socialise with each other. These meetings could be led by the GBMSM themselves and not by a counsellor.  Through mass or social media, and/or health services, suggest GBMSM think the GBMSM-facing organisations as places they can find chances to socialise with other men instead of thinking them as places they can only have the chance for 1 to 1 contact with a professional. |
| **Barriers** |  |  |  |  |  |
| GBMSM find it hard to seek help for gay related MH issues in GBMSM-facing organisations **because GBMSM do not know about these organisations.** | Psychological capability | Knowledge | Education | 5.6 Information about emotional consequences  7.1 Prompts/Cues  6.3 Information about other’s approval | Through mass or social media, and/or health services inform GBMSM about the existence of GBMSM-facing organisations improving GBMSM’s MH.  In social media and gay apps, notifications will appear informing GBMSM about local GBMSM-facing organisations (GPS led).  In social media and gay apps, notifications will appear informing GBMSM about ways of contacting local GBMSM-facing organisations. |
| GBMSM find it hard to seek help for gay related MH issues in GBMSM-facing organisations **because they believe these GBMSM-facing organisations focus mainly on sexual health issues** | Reflective motivation | Environmental context and resources  Professional/social role and identity | Persuasion | 13.2  Framing/ Reframing  5.3 Information about social and environmental consequences  13.1 Identification of self as a role model | Work closely with GBMSM-facing organisations to suggest they rebrand themselves more clearly as places GBMSM improve their MH or well-being instead of places that solely focus on sexual health.  Work closely with GBMSM-facing organisations to show that if they change the focus to include MH issues, that may be a good example for other GBMSM-facing organisations. |
| GBMSM find it hard to seek help for gay related MH issues in GBMSM-facing organisations **because they lack the confidence to search for GBMSM-facing organisations** | Reflective motivation | Beliefs about capabilities | Persuasion  Modelling | 15.3 Focus on past success  6.1 demonstration of the behaviour | Using a range of appropriate media to encourage GBMSM to reflect on the occasions that they managed to search successfully for services and draw attention to how similar searching for culturally appropriate MH care may be.  Using a range of appropriate media show examples of how they could search for local GBMSM-facing organisations that meet their requirements. |
| GBMSM find it hard to seek help for gay related MH issues in GBMSM-facing organisations **because they ‘don’t feel like’ asking for help** | Reflective motivation | Intentions | Education  Persuasion | 5.6 Information about emotional consequences  6.3 information about other’s approval  16.2 Imaginary reward  13.5 identity associated with changed behaviour  13.3 Incompatible beliefs | Using a range of appropriate media explain the positive impact of using GBMSM-facing organisations upon GBMSM’s MH.  Using a range of appropriate media show the number of GBMSM seeking and receiving help through GBMSM-facing organisations.  Using a range of appropriate media circulate messages encourage GBMSM to imagine the psychological benefits they will experience after they receive help from GBMSM-facing organisations.  Using a range of appropriate media ask GBMSM to think themselves as someone who asks for help.  Using a range of appropriate media draw attention to GBMSM’s refusal to ask for help and contrast it with their potential advice to a peer who is should be seeking support for their MH. |
| GBMSM find it hard to seek help for gay related MH issues in GBMSM-facing organisations because **GBMSM do not recognise MH support/counselling as beneficial** | Reflective motivation | Beliefs about consequences | Education  Persuasion | 5.6 Information about emotional consequences  6.3 Information about other’s approval  5.3 Information about social and environmental consequences | Using a range of appropriate media explain the positive impact of using GBMSM-facing organisations upon GBMSM’s MH.  Using a range of appropriate media circulate messages that show the number of GBMSM who found the help they got through GBMSM-facing organisations beneficial. |

| **Table A3.** Synthesis of findings and recommendations. | | | |
| --- | --- | --- | --- |
| **Quantitative findings** | **Qualitative findings** | **Behaviour change wheel analysis findings** | **Recommendations**  Synthesis of our collective interpretations  Integration and implications  **Short/medium/Long term timing** |
| One quarter report poor or very poor MH since the start of the pandemic |  | Clear signal of need for MH provision.  No indicator more than rest of population here. | Short Term: Mass & Social Media - Advertise existing resources to improve MH. (e.g. apps, websites, helplines, counselling) for GBMSM.  Short Term: Mass & Social Media - Raise awareness of simple/useful MH promotion techniques (e.g. exercise, social interaction, reduce alcohol/drugs, mindfulness etc.) for GBMSM.  Medium Term: Develop GBMSM facing/tailored resources to help men with poor MH.  Medium Term: Contemporary research needed to further understand what factors affect the MH of GBMSM (e.g. impact of minority stress, homophobia, disproportionate substance us) and, critically, how they interact with other health inequalities to produce syndemic ill-health among this group.  Long-term: Create community-development initiatives, drawing on assets-based co-creation approach to enhance MH. |
| One fifth of the sample had used MH resources | MH help-seeking through GBMSM-specific organisations was hindered by a lack of lack of motivation to seek help | Given finding above suggestion of unmet need. | Short Term: **GBMSM & MH services** to raise awareness of existing MH resources.  Short Term: **GBMSM & MH services** to raise awareness of success of existing resources to help improve MH (for GBMSM).  Medium Term: **GBMSM organisations** should adapt outreach activities to promote MH and Wellbeing and ensure their MH services are promoted as widely as possible.  Medium Term: **MH services** should be proactive to reach GBMSM.  Long Term: Advocate importance of MH awareness and maintenance for **all**.  Long Term: Advocate importance of MH awareness and maintenance for GBMSM population given their unique multiple vulnerabilities (syndemics). |
| Men **already suffering from moderate to severe anxiety** more likely to seek help for MH issues since the start of the pandemic |  |  | Short Term: Since those with longstanding MH issues already know how to seek help**, s**ervices should focus on those with new MH problems**.**  Short Term: Mass & Social Media - Advertise existing resources to improve MH. (e.g. apps, websites, helplines, counselling) for GBMSM.  Short Term: Mass & Social Media - Raise awareness of simple/useful MH promotion techniques (e.g. exercise, social interaction, reduce alcohol/drugs, mindfulness etc.) for GBMSM.  Medium Term: Consider innovative ways by which these men already seeking help for MH can share their experience to enable others to also do so  Long Term: Advocate importance of MH awareness and maintenance for all. |
| 1. Men with a past MH **diagnosis** more likely to seek help for MH issues since the start of the pandemic. | 2. MH help seeking through GBMSM specific organisations was hindered by a lack of knowledge around GBMSM-facing organisations. | 3. Through targeted mass and social media interventions, ensure potential service users know that MH services exist within gay-related organisations and provide details of their location and ways of accessing them. | 1. Short Term: Since those with longstanding MH issues already know how to seek help, services should focus on those with new MH problems**.**  1. Long Term: Advocate importance of MH awareness and maintenance for all.  1. Long Term: Advocate importance of MH awareness and maintenance for GBMSM population given their unique multiple vulnerabilities (syndemics)  2. Short term: Ensure **GBMSM facing organisations** advertise MH services they offer within wider promotion of their services.  2. Medium Term: **GBMSM organisations** should adapt outreach activities to promote MH and Wellbeing and ensure their MH services are promoted as widely as possible.  3. Medium term: **Mass & social media** advertise testimonials of use and demonstration of how to access services. |
|  | MH help seeking through GBMSM specific organisations was hindered by a lack of confidence to search for organisations | Through a webinar that is promoted through local media, social media, hook up apps, encourage GBMSM to describe the occasions that they managed to search successfully for services they had never used (e.g. first time they used a sexual health service).  Through targeted mass and social media include demonstrations of how GBMSM can search for GBMSM-facing organisations that meet their requirements. | Medium Term: Frame MH help seeking as normative for GBMSM and positively endorsed.  Medium Term: **Peers, partners, friends & family** can play a role in enhancing GBMSM confidence and self-efficacy in help seeking for (online) MH resources – ‘help a friend’.  Medium Term: Run webinar so **GBMSM MH champions** describe how they successfully searched for, and used, services for the first time.  Medium term: **Mass & social media** advertise testimonials of use and demonstration of how to access services. |
|  | 1. Referral/endorsement by a trusted person enabled help seeking with on-line resources | 2. Consider the use of opinion leaders or influencers endorsing these services and organisations.  3. A wide range of health professionals could endorse the use of high-quality MH apps.  4. Key opinion leaders in the GBMSM communities could endorse the use of high-quality MH apps. | 1. Medium Term: **Peers, partners, friends & family** can play a role in enhancing GBMSM confidence and self-efficacy in help seeking for (online) MH resources – ‘help a friend’. (D)  1. Medium Term: Run webinar so **GBMSM MH champions** describe how they successfully searched for, and used, services for the first time. (D)  1,2. Medium term: **Mass & social media** advertise testimonials of use and demonstration of how to access services. (D)  1,2. Medium Term: **GBMSM Champions** can operate at the organisational (e.g. THT), community (e.g. ScotsBears), professional (GPs, STI clinician), celebrity (Lady Gaga) and individual (e.g. Jim McDonald) level.  1,2,3,4. Medium Term: **GBMSM MH champions** frame MH as an issue for GBMSM, endorse and promote i) MH help seeking, ii) MH resources and organisations, iii) high-quality MH apps, using personal testimonials. |
|  | 1. Provision of individualised care enabled help seeking with on-line resources  2. GBMSM find it hard to seek help for MH issues in online apps because these apps are not individualized/tailored to each person’s needs  3. Lack of person-centred approach was a barrier to help seeking with on-line resources | 4. Work collaboratively with app designers to develop individually tailored MH apps that provide individualised MH support. A considered and rigorous approach to user involvement would enhance this process. | 1,3 Medium term: **GBMSM and MH organisations** coproduce MH materials with **GBMSM** **community**.  1-4. Medium Term: **Research, MH and GBMSM organisations and communities** work collaboratively to identify those high-quality MH apps (already identified in systematic reviews) most suitable for GBMSM.  1-4 Medium Term: **GBMSM-facing organisations** endorse and promote those high-quality MH apps suitable to GBMSM, via outreach work, social & mass media.  1,3 Medium Term: After quality appraisal, **GBMSM and MH services** advocate relevance and utility of specific GBMSM-appropriate MH resources. (D)  1,3 Medium Term: After quality appraisal, **GBMSM champions** advocate relevance utility of specific GBMSM-appropriate MH resources (D)  1-4. Medium Term: **Research, GBMSM/MH Organisations and GBMSM community** work together to design GBMSM-tailored MH resources (e.g. meditation programme) using existing modifiable apps (e.g. InsightTimer).  4. Long Term: Collaborative research with **GBMSM communities, MH & GBMSM organisations and app designers** to coproduce MH app tailored to GBMSM.  4. Long Term: RCT to evaluate the efficacy of MH app tailored to GBMSM. |
|  | 1 Clear, concise, & discreet content and function enabled help seeking with on-line resources | 2 Work collaboratively with App designers to develop individually tailored MH apps that provide individualised MH support. A considered and rigorous approach to user involvement would enhance this process. | 1. Medium term: **GBMSM and MH organisations** coproduce MH materials with **GBMSM** **community**.  *2. As above, only those issues where #4 is mentioned.* |
|  | 1 Attitudes towards self-resourcefulness could be barrier to help seeking with on-line resources | 2 Targeted mass and social media could be recommended.  3 Materials should be co-produced with community members. Key messages could include:-  4 The branding of quality apps as self-help rather than as an independent source of help.  5 The positive articulation of a new and valued identity as someone who engages with MH apps to actively maintain their HM.  6 Contrasting GBMSM’s rejection of MH apps with their commitment to improving MH and endorsement of other apps (e.g. social and sociosexual media). | 1,2 Short Term: Mass & Social Media - Advertise existing resources to improve **MH**. (e.g. apps, websites, helplines, counselling) **for GBMSM**.  1,2 Short Term: Mass & Social Media - Raise awareness of simple/useful MH promotion techniques (e.g. exercise, social interaction, reduce alcohol/drugs, mindfulness etc.) **for GBMSM.**  1,2 Short Term: GBMSM & MH services to raise awareness of existing MH resources  1,2 Short Term: GBMSM & MH services to raise awareness of success of existing resources to help improve MH (for GBMSM).  1,2 Medium term: **Mass & social media** advertise testimonials of use and demonstration of how to access services.  3 Medium term: **GBMSM and MH organisations** coproduce MH materials with **GBMSM** **community**.  3,4 Long Term: Collaborative research with **GBMSM communities, MH & GBMSM organisations and app designers** to coproduce MH app tailored to GBMSM.  4,5. Medium Term: **GBMSM MH champions** frame MH as an issue for GBMSM, endorse and promote i) MH help seeking, ii) MH resources and organisations, iii) high-quality MH apps, using personal testimonials.  4,5 Medium Term: **GBMSM-facing organisations** endorse and promote those high-quality MH apps suitable to GBMSM, via outreach work, social & mass media.  4,5 Medium Term: **Research, GBMSM/MH Organisations and GBMSM community** work together to design GBMSM-tailored MH resources (e.g. meditation programme) using existing modifiable apps (e.g. InsightTimer).  4,5. Medium Term: Local **GBMSM services / community champions** provide support in using the apps (LGBT MH app support group, training intervention).  5 Long Term: Create new identity of MH maintainer (like exercising, 5-a-day etc.) for GBMSM as someone who actively engages in activities to maintain their MH (e.g. exercise, meditation etc.).  6. Medium Term: Promote MH maintenance as a long-term commitment (i.e. Ruby Wax ‘deposit in saving bank’) not a ‘quick fix’ (i.e. one session of meditation unlikely to make you feel better.  6. Medium term: NHS/GBMSM organisations devise strategies to ensure digitally excluded GBMSM are included when services are delivered remotely. |
|  | 1. Feedback about a positive effect on MH enabled help seeking with on-line resources  2. GBMSM find it easy to seek help for MH issues from online apps **because they find that using these apps have a direct *positive* effect upon their MH**  3 Lack of perceived benefit was a barrier to help seeking with on-line resources  4 MH help seeking through GBMSM specific organisations was hindered by perceptions that counselling was not beneficial | 5 If there is enough evidence to support it, and MH Apps can be endorsed with confidence, then consider a suite of educational and persuasive resources that use targeted mass and social media could be recommended. Materials should be co-produced with community members. Key messages could include:-  6 Inform GBMSM about the number and types of GBMSM who have benefited from MH apps if this available  7 Inform GBMSM about the beneficial effect of MH apps upon GBMSM’s MH if this is proven  8 Through targeted mass and social media interventions ensure potential service users know that MH services are beneficial and can contribute to improved health and wellbeing.  9 Consider the inclusion of key opinion leaders endorsing the use of MH services including personal testimonials from service users | 1-4 Short Term: Mass & Social Media – Advertise existing resources to improve MH. (e.g. apps, websites, helplines, counselling) for GBMSM.  1-4 Short Term: Mass & Social Media – Raise awareness of simple/useful MH promotion techniques (e.g. exercise, social interaction, reduce alcohol/drugs, mindfulness etc.) for GBMSM.  1-4 ,7, 8 Short Term: GBMSM & MH services to raise awareness of success of existing resources to help improve MH (for GBMSM).  5 Short Term: Working with national, regional and local stakeholders to consider the cocreation of a GBMSM population targeted mass and social media intervention to reach diverse GBMSM.  4 Short term: Ensure GBMSM facing organisations advertise MH services they offer within wider promotion of their services  1-5 Medium Term: **Research, MH and GBMSM organisations and communities** work collaboratively to identify those high-quality MH apps (already identified in systematic reviews) most suitable for GBMSM.  1-5, 7 Medium Term: After quality appraisal, **GBMSM and MH** services advocate relevance and utility of specific GBMSM-appropriate MH resources.  1-4, 9 Medium Term: After quality appraisal, **GBMSM champions** advocate relevance and utility of specific GBMSM-appropriate MH resources  9 Medium Term: **GBMSM MH champions** frame MH as an issue for GBMSM, endorse and promote i) MH help seeking, ii) MH resources and organisations, iii) high-quality MH apps, using personal testimonials.  1, 3 7, 9. Medium Term: **Peers, partners, friends & family** can play a role in enhancing GBMSM confidence and self-efficacy in help seeking for (online) MH resources – ‘help a friend’.  1-4, 7 Medium Term: Frame MH help seeking and self-help use of high-quality MH app use as normative for GBMSM and positively endorsed.  Long-term: Create community-development initiatives, drawing on assets-based co-creation approach to enhance MH  Long Term: Advocate importance of MH awareness and maintenance for all.  Long Term: Advocate importance of MH awareness and maintenance for GBMSM population given their unique multiple vulnerabilities (syndemics). |
|  | MH help seeking through GBMSM specific organisations was hindered by perceptions they only dealt with sexual health | A range of organisations offering gay-related services should be made aware of the prevalence and need for expanded MH services  Organisations should be encouraged to examine their mission statement and values and potentially reconsider their branding if they offer both sexual and MH services  Commissioners of MH services should work closely with organisations offering wider gay-related services and ensure that MH pathways are clear and accessible. Aspects of the visibility of MH pathways could form part of a service level agreement.  Demonstrate to GBMSM-facing organisations counsellors/advisors how to tackle MH issues with GBMSM via a role-play exercise. | Medium Term: Rebranding of **GBMSM organisations** to include MH issues. Moving away from GBMSM facing organisations due to ending HIV.  Medium Term: Work with **GBMSM** and **lobby boards** for MH inclusion.  Medium Term: Connect **MH with SH** and service use more broadly (holistic health).  Medium Term: **GBMSM-facing services** develop and deliver GBMSM staff training package for generic MH and wider services.  Long Term: Work to ensure **generic MH organisations** dealing with specific issues (alcohol, drugs, abuse, eating disorders, MH etc.) are gay friendly. |
|  | MH help seeking through GBMSM specific organisations was enabled through safe, inclusive and discrete services | Ensure resources pertaining to MH amongst GBMSM are available in discrete formats (e.g. smaller contact cards, private Zoom calls). | Medium term: **GBMSM and MH organisations** coproduce MH materials with **GBMSM** **community**.  Medium Term: Communicate these issues via a webinar and linked short briefing in order to ensure consistency across the sector. Primary focus is to embed good practice within service delivery. |
|  | MH help seeking through GBMSM specific organisations was enabled through having diverse counsellors (sexual identity and racialised status) who expressed empathy | Enhance and celebrate the visibility of diverse and inclusive organisations that meet the needs of highly varied service users (e.g. including trans and non-binary).  Ensure gay related organisations that offer MH services visibly celebrate the diversity of their staff and clients.  Work with diverse organisations and for those that are inclusive of trans and non-binary people make sure that it is understood they may be a good example for other GBMSM-facing organisations s too. | Short Term: Ensure ‘branding’ is inclusive and visibly welcome to all – celebrate diversity of services users and, where possible, staff.  Medium term: **GBMSM and MH organisations** coproduce MH materials with **GBMSM** **community**.  Long term: National level service provision (e.g. Scotland-wide, UK-wide) could enable celebration of diversity of service provider staff more realistically.  Medium Term: Communicate these issues via a webinar and linked short briefing in order to ensure consistency across the sector. Primary focus is to embed good practice within service delivery.  This work is already under way in certain sectors (e.g. HIV Scotland, Bissell, S; McKenna, R 2020; Alcohol Dependency Services work, Emslie et al 2021) |
|  | MH help seeking through GBMSM specific organisations was enabled by providing a safe, empathic, & comfortable environment that included opportunities for socialisation with other gay men | Ensure that gay related organisations offering MH services are reassuring potential service users that the environment is safe and comfortable.  Ensure that gay facing organisations offering MH services are actively monitoring and improving the safety and comfort of their service through ongoing service improvement. | Medium term: **GBMSM and MH organisations** coproduce MH materials with **GBMSM** **community**.  Medium term: Important to think of social and discrete spaces for both **physical and digital services**.  Medium Term: Communicate these issues via a webinar and linked short briefing in order to ensure consistency across the sector. Primary focus is to embed good practice within service delivery. |
|  | GBMSM find it easy to seek help for gay related MH issues in gay-related organisations **because they receive counselling from counsellors with empathy** | Ensure those providing MH services to GBMSM are maintaining their competencies particularly in regard to empathy and listening skills. | Medium Term: **GBMSM-facing services** develop and deliver GBMSM staff training package for generic MH and wider services.  Long Term: Work to ensure **generic MH organisations** dealing with specific issues (alcohol, drugs, abuse, eating disorders, MH etc.) are gay friendly.  This work is already under way in certain sectors (e.g. HIV Scotland, Bissel, S; McKenna, R 2020; Alcohol Dependency Services work, Emslie et al 2021)  Medium Term: Communicate these issues via a webinar and linked short briefing in order to ensure consistency across the sector. Primary focus is to embed good practice within service delivery. |
|  | GBMSM find it hard to seek help for gay related MH issues in gay-related organisations **because they ‘don’t feel like’ asking for help** | Through targeted mass and social media interventions ensure potential service users know that MH services are beneficial and can contribute to improved health and wellbeing  Key messages could focus on depicting people who need support not thinking that they do  Draw attention to the contrast between how an intervention recipient might feel (e.g. I’m not asking for help) with what they would say to their best friend (e.g. You should seek help even if you don’t want to ask for it). | Medium Term: Frame MH help seeking as normative for GBMSM and positively endorsed.  Long Term: Advocate importance of MH awareness and maintenance for all.  Long Term: Advocate importance of MH awareness and maintenance for GBMSM population given their unique multiple vulnerabilities (syndemics)  Long Term: Create new identity of MH maintainer (like exercising, 5-a-day etc.) for GBMSM as someone who actively engages in activities to maintain their MH (e.g. exercise, meditation etc.).  Medium term: **Mass & social media** advertise testimonials of use and demonstration of how to access services.  Medium Term: **Peers, partners, friends & family** can play a role in enhancing GBMSM confidence and self-efficacy in help seeking for (online) MH resources – ‘help a friend’. |
